# Interleukin enhancer-binding factor 3 and HOXC8 co-activate cadherin 11 transcription to promote breast cancer cells proliferation and migration

**DOI:** 10.18632/oncotarget.22491

**Published:** 2017-11-18

**Authors:** Yang Zhang, Chenchen Yang, Mingsheng Zhang, Houli Liu, Chen Gong, Jie Zhang, Shanshan Xu, Jin Zou, Yuanzhong Kai, Yong Li

**Affiliations:** ^1^ School of Life Sciences, Anhui University, Hefei, Anhui Province, China; ^2^ Department of Oncology, Tongji Hospital, Tongji Medical College, Huazhong University of Science and Technology, Wuhan, Hubei, China

**Keywords:** CDH11, ILF3, HOXC8, transcriptional regulation, breast cancer

## Abstract

Cadherin 11 (CDH11) expression is detected only in invasive breast cancer cells and aggressive breast cancer specimens. However, little is known about the molecular mechanisms of CDH11 transcriptional regulation. Here, we report that interleukin enhancer binding factor 3 (ILF3) interacts with Homeobox C8 (HOXC8) to activate CDH11 transcription in breast cancer cells. Using co-immunoprecipitation and mass spectrometry analyses, ILF3 is shown to interact with HOXC8 in breast cancer cells. We demonstrate that ILF3 binds to the CDH11 promoter on nucleotides –2982 ~ –2978 and –2602 ~ 2598 and interacts with HOXC8 to co-activate CDH11 transcription. We further show that ILF3 promotes proliferation and migration, at least partially, by facilitating CDH11 expression in breast cancer cells. Moreover, immunohistochemistry (IHC) shows that expression of CDH11, ILF3 and HOXC8 are all upregulated in breast cancer specimens compared to normal breast tissues. Importantly, the expression levels of CDH11, ILF3 and HOXC8 are elevated in the advanced stages of breast cancer, and high expression of CDH11, ILF3 and HOXC8 is associated with poor distant metastasis-free survival (DMFS) for breast cancer patients.

## INTRODUCTION

The cadherin switch, which is an increase in the expression of N-cadherin and/or cadherin 11 and a decrease in E-cadherin, is associated with both the epithelial-to-mesenchymal transition (EMT) and cancer progression [1, 2]. Cadherin 11 (CDH11), also known as OB-cadherin, was originally identified in mouse osteoblasts and is expressed predominantly in mesenchymal tissues [3, 4]. CDH11 expression has been detected in multiple types of cancer including breast cancer, prostate cancer, colon cancer, gastric cancer, renal cell carcinoma, and osteosarcoma [5–10]. In breast cancer, CDH11 is expressed only in invasive cell lines and aggressive clinical specimens, and ectopically expressing CDH11 enhances the migration and metastasis of breast cancer cells, suggesting that CDH11 plays an important role in breast cancer progression and metastasis [5, 11, 12]. Therefore, defining the regulatory mechanisms of CDH11 expression may unveil more effective therapeutic strategies to control breast cancer progression and metastasis.

We previously reported that Homeobox C8 (HOXC8) acts as a transcription factor to induce CDH11 expression in breast cancer cells [11, 13]. HOXC8 belongs to the 39-member Homeobox (HOX) family and participates in multiple physiological processes, such as cell proliferation, migration, adhesion, and differentiation [14]. Dysfunction of HOXC8 is implicated in various cancers, including breast, prostate, cervical and pancreatic cancers, by facilitating cell migration and metastasis [12, 15–17]. All HOX (homeobox) genes encode homeodomain-containing transcription factors and associate with other protein factors to regulate the expression of multiple genes [18, 19]. To further clarify the roles of HOXC8 in regulation of CDH11 transcription in breast cancer cells, we immunoprecipitated HOXC8 protein complexes and observed that interleukin enhancer-binding factor 3 (ILF3) co-precipitated with the HOXC8 protein in this study.

The interleukin-enhancer binding factor 3 (ILF3) family members include NF90a, NF90b, NF110a and NF110b, and all of them are splicing variants of ILF3 transcripts [20–22]. All members share a long and identical N-terminal domain but differ with specific C-terminal domains [20]. ILF3 proteins are implicated in a variety of cellular processes, including DNA metabolism, transcription, translation, RNA stability and microRNA biogenesis [23–27]. Moreover, it has been reported that ILF3 plays an important role in tumorigenesis of multiple cancers, such as hepatocellular carcinoma, non-small cell lung carcinoma, breast cancer and ovarian cancer [28–31].

In this study, we demonstrated that CDH11 transcription required the formation of a protein complex containing the HOXC8, NF90 and NF110 proteins in breast cancer cells. Using co-immunoprecipitation (Co-IP) and mass spectrometry analyses (MS), we observed that ILF3 co-purified with HOXC8 and co-localized with HOXC8 in the nuclei of breast cancer cells. We further demonstrated that ILF3 bound to CDH11 promoter to co-activate CDH11 transcription with HOXC8. Moreover, we found that ILF3 increased proliferation and migration of breast cancer cells by facilitating CDH11 expression. Lastly, we showed that CDH11, ILF3 and HOXC8 were expressed at statistically higher levels in breast cancer specimens than in normal breast tissues, and the association of CDH11, ILF3 and HOXC8 was linked to poor distant metastasis-free survival for breast cancer patients.

## RESULTS

### ILF3 interacts with HOXC8 in Hs578T and MDA-MB-231 cells

**Figure 1:**
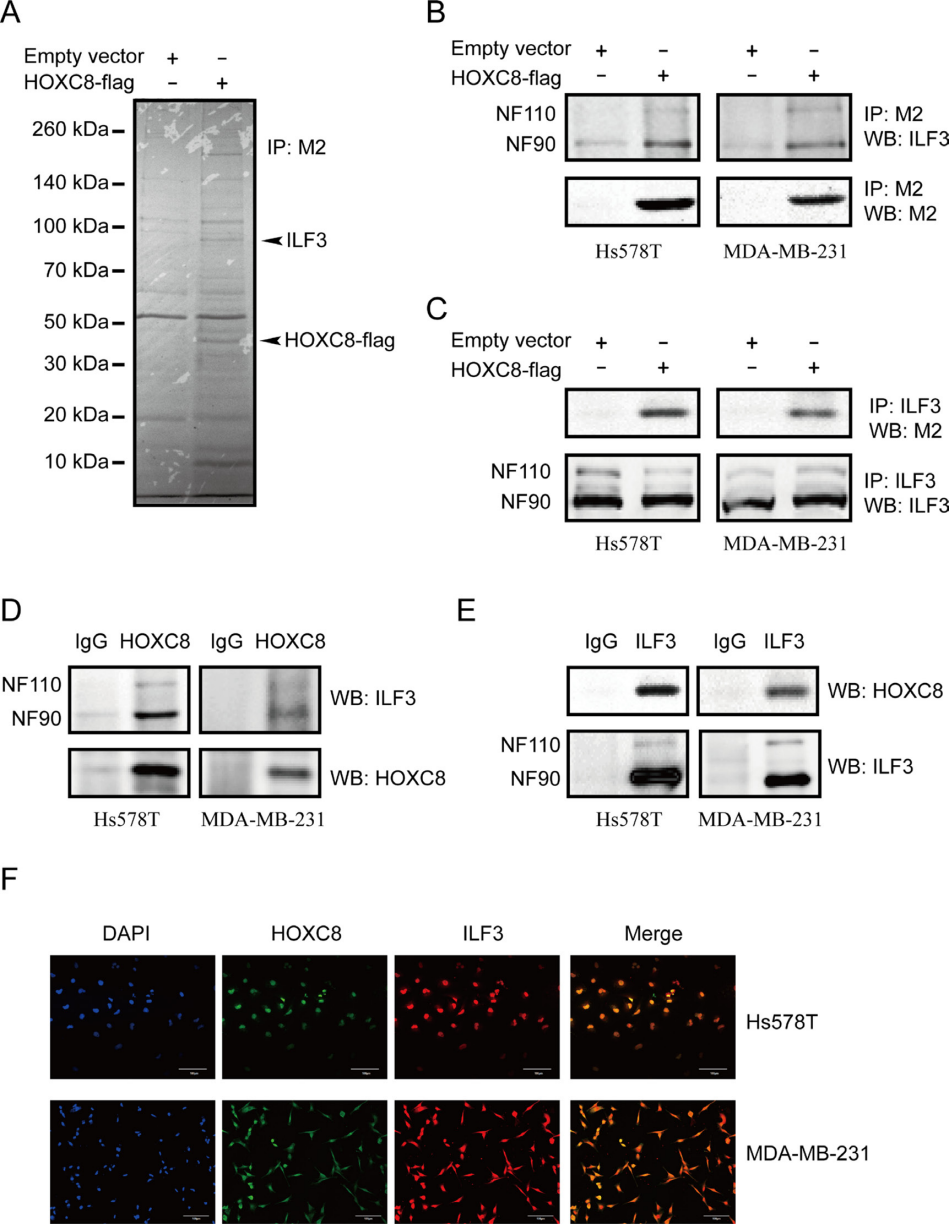
ILF3 interacts with HOXC8 in breast cancer cells (**A**) MDA-MB-231 cells were lentivirally transduced with the empty vector or vector encoding HOXC8-flag. Co-immunoprecipitations performed with the anti-flag M2 antibody were separated by SDS–PAGE, and the differential bands were analyzed by MS. (**B**) Co-immunoprecipitation (Co-IP) was performed using the M2 antibody in Hs578T or MDA-MB-231 cells that were lentivirally transduced with the empty vector or vector encoding HOXC8-flag. Immunoprecipitates were analyzed by Western blotting (WB). (**C**) Co-IP was performed using the ILF3 antibody in Hs578T or MDA-MB-231 cells that were lentivirally transduced with the empty vector or vector encoding HOXC8-flag. Immunoprecipitates were analyzed by WB. (**D**) Co-IP experiment was performed using anti-HOXC8 antibody in Hs578T or MDA-MB-231 cells, and precipitated proteins were analyzed by WB using anti-ILF3 or anti-HOXC8 antibodies as indicated. (**E**) Co-IP experiment was performed using anti-ILF3 antibody in Hs578T or MDA-MB-231 cells, and precipitated proteins were analyzed by WB using anti-HOXC8 or ILF3 antibodies as indicated. (**F**) Immunofluorescence staining for HOXC8-flag (green) and ILF3 (red) in Hs578T or MDA-MB-231 cells that were lentivirally transduced with the vector encoding HOXC8-flag; DAPI staining for the nucleus. Magnification, 200×; Scale bar, 100 μm.

In our previous study, we found that HOXC8 bound to CDH11 promoter to activate CDH11 transcription. To further elucidate the regulatory mechanisms of CDH11 transcription, we performed HOXC8 co-immunoprecipitation experiments to identify proteins that interacted with the HOXC8 protein. MDA-MB-231 cells were lentivirally transduced with HOXC8-flag expression vectors or empty vectors, and immunoprecipitation was performed using anti-Flag M2 antibodies. Mass spectrometry analyses revealed that ILF3 proteins were co-precipitated with the HOXC8 protein (Figure 1A).

To confirm the mass spectrometry data, co-immunoprecipitation assays of HOXC8 and ILF3 were performed in Hs578T and MDA-MB-231 cells that both expressed CDH11 protein. As shown in Figure 1B, we found that both NF90 and NF110 co-precipitated with HOXC8-flag protein. We next carried out ILF3 Co-IP experiments and found that HOXC8-flag protein co-precipitated with ILF3 in both Hs578T and MDA-MB-231 cells (Figure 1C). This observation was confirmed by Co-IP of endogenous HOXC8 and ILF3 proteins in both Hs578T and MDA-MB-231 cells, suggesting that endogenous HOXC8 has interaction with endogenous ILF3 proteins, too (Figure 1D and 1E). To validate the interaction between ILF3 and HOXC8, immunofluorescence analyses were carried out in Hs578T and MDA-MB-231 cells. As shown in Figure 1F and Supplementary Figure 1, ILF3 and HOXC8-flag proteins were found to co-localize within nuclei of Hs578T or MDA-MB-231 cells by confocal microscopy. Taken together, these data indicated that ILF3 interacted with HOXC8 in CDH11-expressing breast cancer cell lines.

### ILF3 is required for CDH11 expression

**Figure 2:**
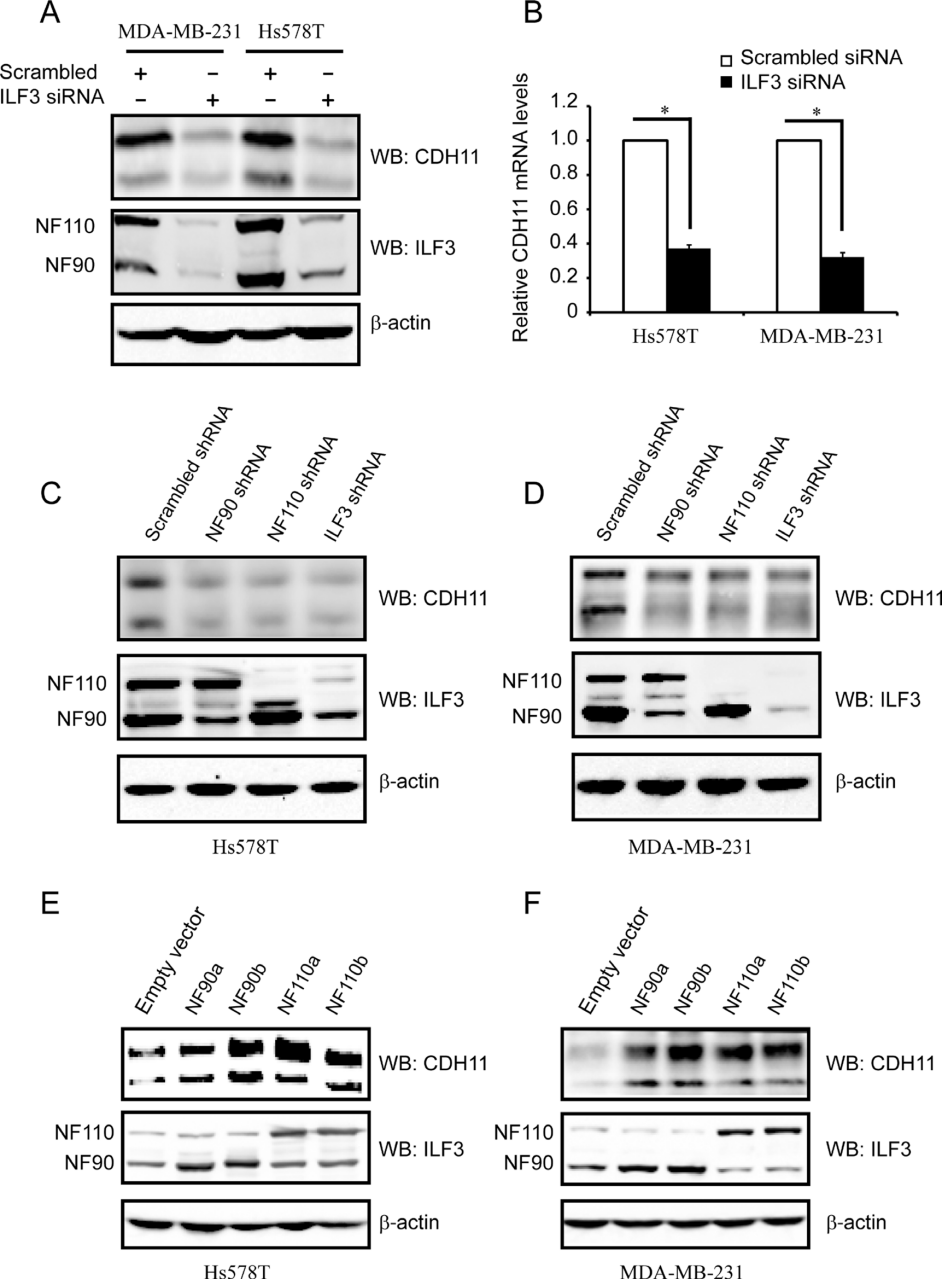
ILF3 activates CDH11 expression in the Hs578T and MDA-MB-231 cell lines (**A**) Hs578T and MDA-MB-231 cells were transfected with siRNAs that specifically targeted ILF3 or with scrambled siRNAs. Cell lysates were immunoblotted for CDH11, ILF3 and β-actin, as indicated. (**B**) Hs578T and MDA-MB-231 cells were transfected with siRNAs that specifically targeted ILF3 or with scrambled siRNAs. Total RNA was subjected to qRT-PCR to examine the levels of CDH11 mRNA. β-actin mRNA was used as an internal control for standardization. (**C** and **D**) Hs578T or MDA-MB-231 cells were lentivirally transduced with NF90-, NF110- or ILF3-specific shRNA, and Western blotting was performed with antibodies for CDH11, ILF3 or β-actin, as indicated. (**E** and **F**) Hs578T or MDA-MB-231 cells were lentivirally transduced with NF90a, NF90b, NF110a or NF110b expression vectors, and Western blotting was performed with antibodies for CDH11, ILF3 or β-actin, as indicated.

Given that ILF3 interacts with HOXC8, we assumed that ILF3 was probably involved in HOXC8-regulated CDH11 transcription. To test this hypothesis, we first examined CDH11 protein and mRNA levels in ILF3-knockdown cells. As shown in Figure 2A and 2B, depletion of ILF3 resulted in significant reduction in CDH11 protein and mRNA levels in Hs578T and MDA-MB-231 cells. Since ILF3 family contains NF90a, NF90b, NF110a and NF110b and the designed ILF3 siRNAs target all splicing isoforms of ILF3, we examined CDH11 expression in NF90 or NF110 shRNA knockdown cells [32]. Immunoblotting showed that knockdown of NF90, NF110 or ILF3 led to a significant reduction in CDH11 protein expression in both Hs578T and MDA-MB-231 cells (Figure 2C and 2D), which was consistent with the observation that silencing of NF90, NF110 or ILF3 resulted in a decrease of about 60% in CDH11 mRNA levels (Supplementary Figure 2). These data indicated that both NF90a/b and NF110a/b were involved in the regulation of CDH11 expression.

To determine which member of ILF3 family was involved in the regulation of CDH11 expression, we examined CDH11 expression by lentivirally transducing breast cancer cells with expression vectors encoding NF90a, NF90b, NF110a or NF110b. Immunoblotting and qRT-PCR assays showed that forced expression of each ILF3 member significantly enhanced CDH11 mRNA and protein levels in both Hs578T and MDA-MB-231 cells (Figure 2E, 2F and Supplementary Figure 3). Taken together, these data indicated that all ILF3 family members were involved in the regulation of CDH11 expression in breast cancer cells.

### ILF3 binds to CDH11 promoter to activate CDH11 transcription

**Figure 3:**
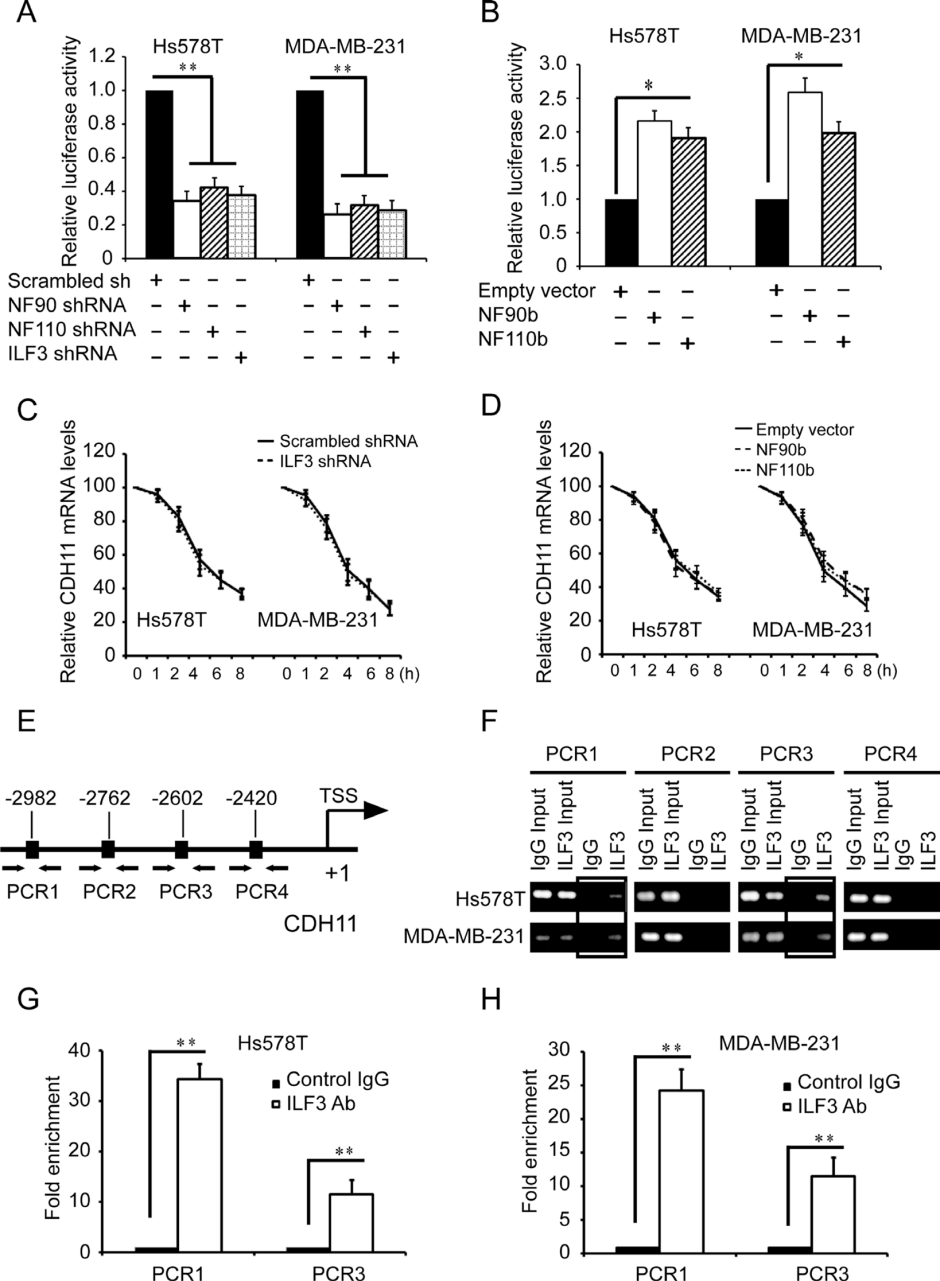
ILF3 binds to CDH11 promoter and activates CDH11 transcription in breast cancer cells (**A**) Luciferase assays were performed in cells transduced with CDH11 promoter luciferase reporter vectors in NF90-, NF110- or ILF3-specific shRNA knockdown cells. The luciferase activity was normalized to the Renilla activity. (**B**) Luciferase assays were performed in cells transduced with CDH11 promoter luciferase reporter vectors in NF90b or NF110b ecto-expression cells. (**C**) Control or ILF3-knockdown cells were treated with 1 μg/ml actinomycin D for varying times (0, 1, 2, 4, 6 and 8 h). Total RNA was extracted and then subjected to qRT-PCR to analyze the level of CDH11 mRNA. β-actin mRNA was used as the internal standard. (**D**) Cells transduced with vectors encoding NF90b, NF110b or empty vectors were treated with 1μg/ml actinomycin D for varying times (0, 1, 2, 4, 6 and 8 h). Total RNA was extracted and then subjected to qRT-PCR to analyze the level of CDH11 mRNA. The level of β-actin mRNA was used as the internal standard. (**E**) schematic diagram of the positions of the putative ILF3 binding sites on the CDH11 promoter. Arrows show the regions for PCR primer amplification. (**F**) ChIP was performed using the ILF3 antibody, and the immunoprecipitated chromatin DNA was subjected to PCR. (**G** and **H**) ChIP was performed using the ILF3 antibody, and the immunoprecipitated chromatin DNA was subjected to real-time PCR. Each sample was run in triplicate and in multiple experiments for mean ± SEM; ^*^P < 0.05; ^**^P < 0.01.

Studies showed that ILF3 participated in not only transcription but also RNA stability [23, 25], so we explored the effects of ILF3 on both CDH11 transcription and mRNA stability. Luciferase analyses using CDH11 promoter luciferase reporter plasmids showed that silencing NF90, NF110 or ILF3 resulted in significant decreases in the luciferase activities (Figure 3A), while expressing NF90 or NF110 greatly enhanced the luciferase activities compared to the negative control (Figure 3B). In actinomycin chase experiments, knockdown of ILF3 did not affect the half-life of CDH11 mRNA (Figure 3C), which was consistent with the ecto-expression of NF90 or NF110 in both Hs578T and MDA-MB-231 cells (Figure 3D). These experiments indicated that ILF3 regulated CDH11 transcription.

Based on reported ILF3 protein binding consensus sequences CTGTT [23], we analyzed the sequence of CDH11 promoter and found four putative ILF3 binding sites on nucleotides –2982 ~ –2978, –2762 ~ –2758, –2602 ~ –2598, and –2420 ~ –2416 of the CDH11 promoter. We designed 4 sets of PCR primers that specifically amplified each region containing the putative ILF3 binding sequence in the CDH11 promoter (Figure 3E) and performed ChIP using anti-ILF3 antibodies to investigate the ILF3 binding sites in CDH11 promoter. ChIP analyses showed that ILF3 bound to CDH11 promoter on nucleotides –2982 ~ –2978 and –2602 ~ 2598, but not on nucleotides –2762 ~ –2758 and –2420 ~ 2416 (Figure 3F, 3G and 3H).

**Figure 4:**
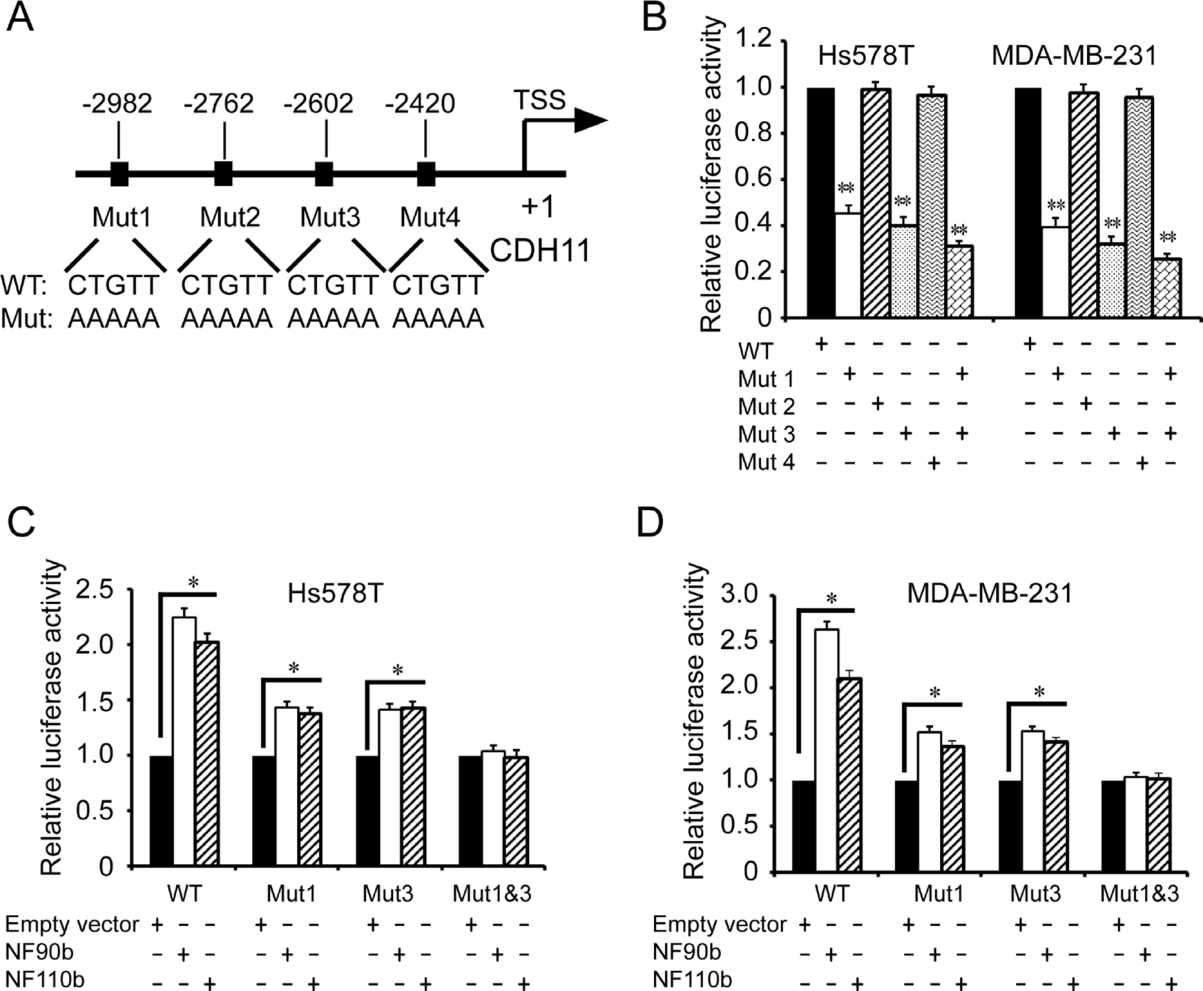
Identification of the ILF3 binding sites in the CDH11 promoter by mutagenesis (**A**) A schematic diagram for the mutagenesis of four CTGTT (putative ILF3 binding) sites in the CDH11 promoter. (**B**) Luciferase analyses were performed with wild-type (WT) or mutant (Mut) CDH11 promoter luciferase reporter vectors in Hs578T or MDA-MB-231 cells. The luciferase activity was measured and normalized to the Renilla activity. Columns, mean; bars, SEM; ^**^P < 0.01. (**C** and **D**) Hs578T or MDA-MB-231 cells were lentivirally transduced with NF90b, NF110b expression vectors or the empty vectors and then transfected with mutant (Mut) CDH11 promoter luciferase reporter vectors, as indicated. The luciferase activity was measured and normalized to the Renilla activity. Each sample was run in triplicate and in multiple experiments for mean ± SEM; ^*^P < 0.05; ^**^P < 0.01.

To further determine the ILF3 binding sites in CDH11 promoter, we performed mutagenesis to mutate these putative ILF3 binding sites (Figure 4A). Luciferase assays showed that only the sequences at the sites of nucleotides –2982 ~ –2978 and –2602 ~ 2598 were responsible for CDH11 promoter activities, while the mutagenesis of the sites of nucleotides –2762 ~ –2758 and –2420 ~ 2416 did not alter CDH11 promoter activities (Figure 4B). This observation was further supported by luciferase analyses performed in ILF3 ecto-expressing cells, in which the mutagenesis of the sites of nucleotides –2982 ~ –2978 and –2602 ~ 2598 abolished the ILF3 effects on CDH11 promoter activities (Figure 4C and 4D). Taken together, these data indicated that ILF3 bound to CDH11 promoter at the sites of nucleotides –2982 ~ –2978 and –2602 ~ 2598 and functioned as a transcriptional activator to regulate CDH11 transcription in breast cancer cell lines.

### ILF3 and HOXC8 bind to CDH11 promoter to cooperatively activate its transcription

**Figure 5:**
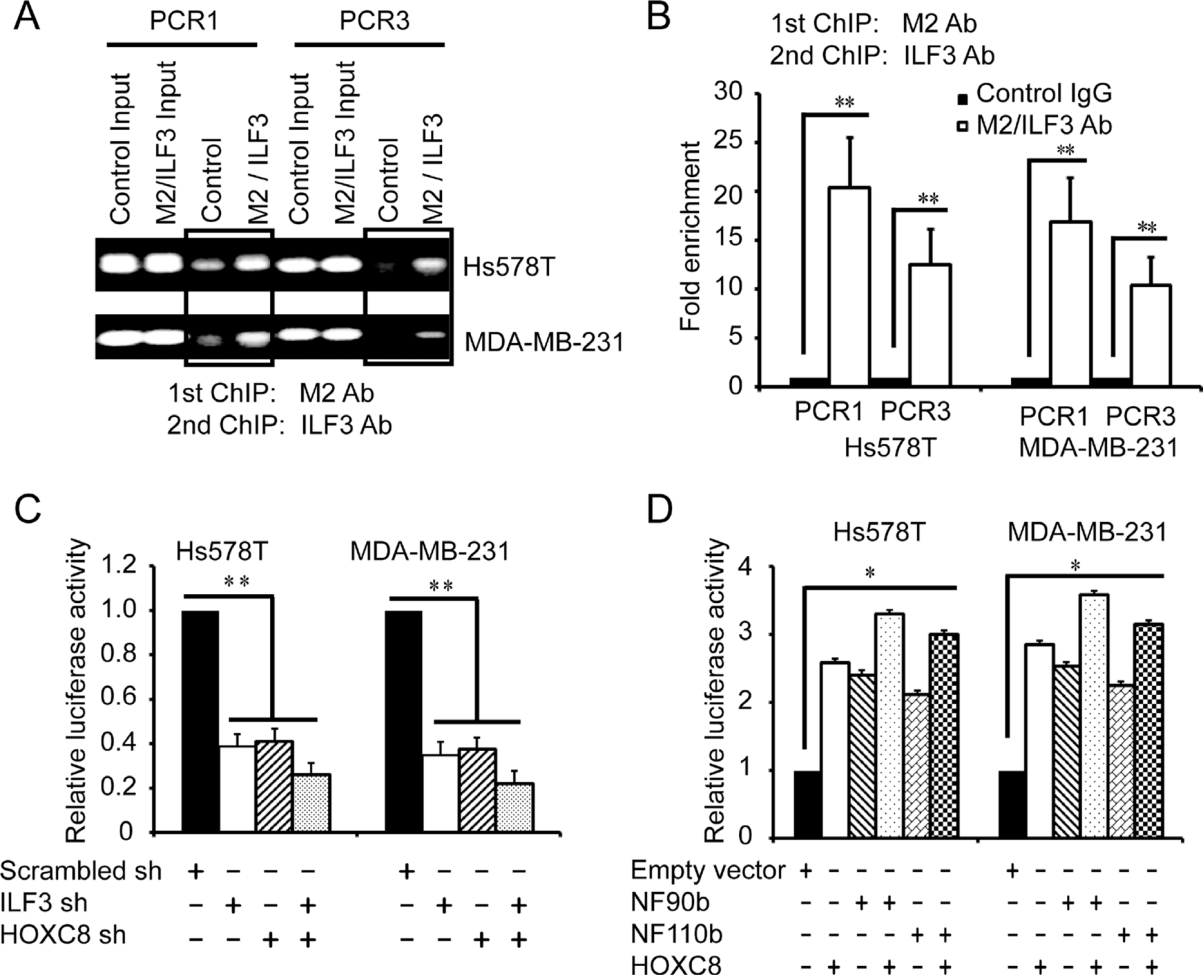
ILF3 and HOXC8 co-occupy the CDH11 promoter to activate CDH11 transcription (**A** and **B**) Sequential ChIP assays were performed in Hs578T or MDA-MB-231 cells that were lentivirally transduced with HOXC8-flag expression vectors. Chromatin was incubated with the anti-flag M2 antibody, and the immunocomplexes were subjected to a second round of ChIP using antibodies against ILF3 or with rabbit IgG as the control. Precipitated chromatin DNA was subjected to PCR or real-time PCR. (**C**) Cells were lentivirally transduced with ILF3 shRNA, HOXC8 shRNA or scrambled shRNA vectors as indicated and transfected with CDH11 promoter luciferase reporter plasmids. The luciferase activity was measured and normalized to the Renilla activity. (**D**) Cells were lentivirally transduced with vectors encoding NF90b, NF110b or HOXC8 and transfected with CDH11 promoter luciferase reporter plasmids. The luciferase activity was measured and normalized to the Renilla activity. Each sample was run in triplicate and in multiple experiments for mean ± SEM; ^*^P < 0.05; ^**^P < 0.01.

The above observations led us to presume that ILF3 and HOXC8 co-occupied CDH11 promoter to co-regulate CDH11 transcription in breast cancer cells. To explore this, we performed sequential ChIP assays. In the Hs578T and MDA-MB-231 cells that were lentivirally transduced with HOXC8-flag expression vectors or empty vectors, sequential ChIP assays were carried out with anti-flag M2 antibodies and followed with anti-ILF3 antibodies. The results from the sequential ChIP assays showed that ILF3 and HOXC8 co-occupied CDH11 promoter in both the Hs578T and MDA-MB-231 cells (Figure 5A and 5B).

To determine whether ILF3 and HOXC8 co-regulated CDH11 transcription in breast cancer cells, luciferase assays with CDH11 promoter reporter plasmids were performed. As shown in Figure 5C, knockdown of ILF3 reduced the activities of CDH11 promoter compared to the negative control, and comparable results were obtained from HOXC8 knockdown cells. Moreover, double knockdown of ILF3 and HOXC8 led to a stronger reduction in the activities of CDH11 promoter. In addition, ecto-expression of HOXC8, NF90 or NF110 enhanced the activities of CDH11 promoter, and ecto-expression of HOXC8 plus ILF3 resulted in higher transcriptional activities of CDH11 promoter (Figure 5D). Together, these observations indicated that ILF3 and HOXC8 formed a protein complex to co-activate CDH11 transcription in breast cancer cells.

### ILF3 promotes proliferation and migration by regulating CDH11 expression

**Figure 6:**
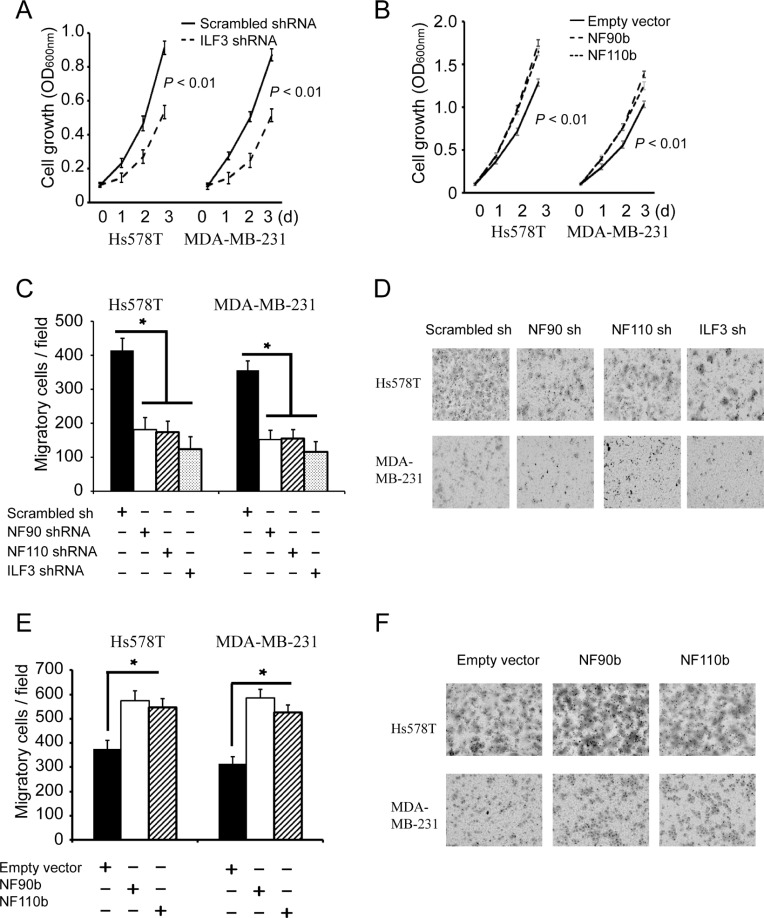
ILF3 promotes proliferation and migration breast cancer cells (**A**) MTT assays to analyze cell proliferation of control, or ILF3 knockdown cells. Data are the mean ± SEM; *n* = 3; *P* < 0.01. (**B**) MTT assays were carried out in cells that were lentivirally transduced with empty vectors or vectors encoding NF90b or NF110b. Data are the mean ± SEM; *n* = 3; *P* < 0.01. (**C** and **D**) Transwell assays to analyze cell migration of control, NF90, NF110 or ILF3 knockdown cells. Columns, mean; bars, SEM; *n* = 3; ^*^P < 0.05. (**E** and **F**) Transwell assays to analyze migration of cells that were lentivirally transduced with empty vectors or vectors encoding NF90b or NF110b. Columns, mean; bars, *n* = 3; SEM; ^*^P < 0.05.

It has been demonstrated that CDH11 promotes breast tumorigenesis [5, 6, 11], so we examined the effects of ILF3 on proliferation and migration of breast cancer cells. MTT assays showed that knockdown of NF90, NF110 or ILF3 significantly decreased breast cell proliferation, and ecto-expression of NF90 or NF110 increased breast cancer cell proliferation (Figure 6A and 6B). Transwell assays further showed that knockdown of NF90, NF110 or ILF3 inhibited migration of breast cancer cells (Figure 6C and 6D), and ecto-expression of NF90 or NF110 promoted migration of breast cancer cells (Figure 6E and 6F). These data indicated that ILF3 promoted proliferation and migration of breast cancer cells.

**Figure 7:**
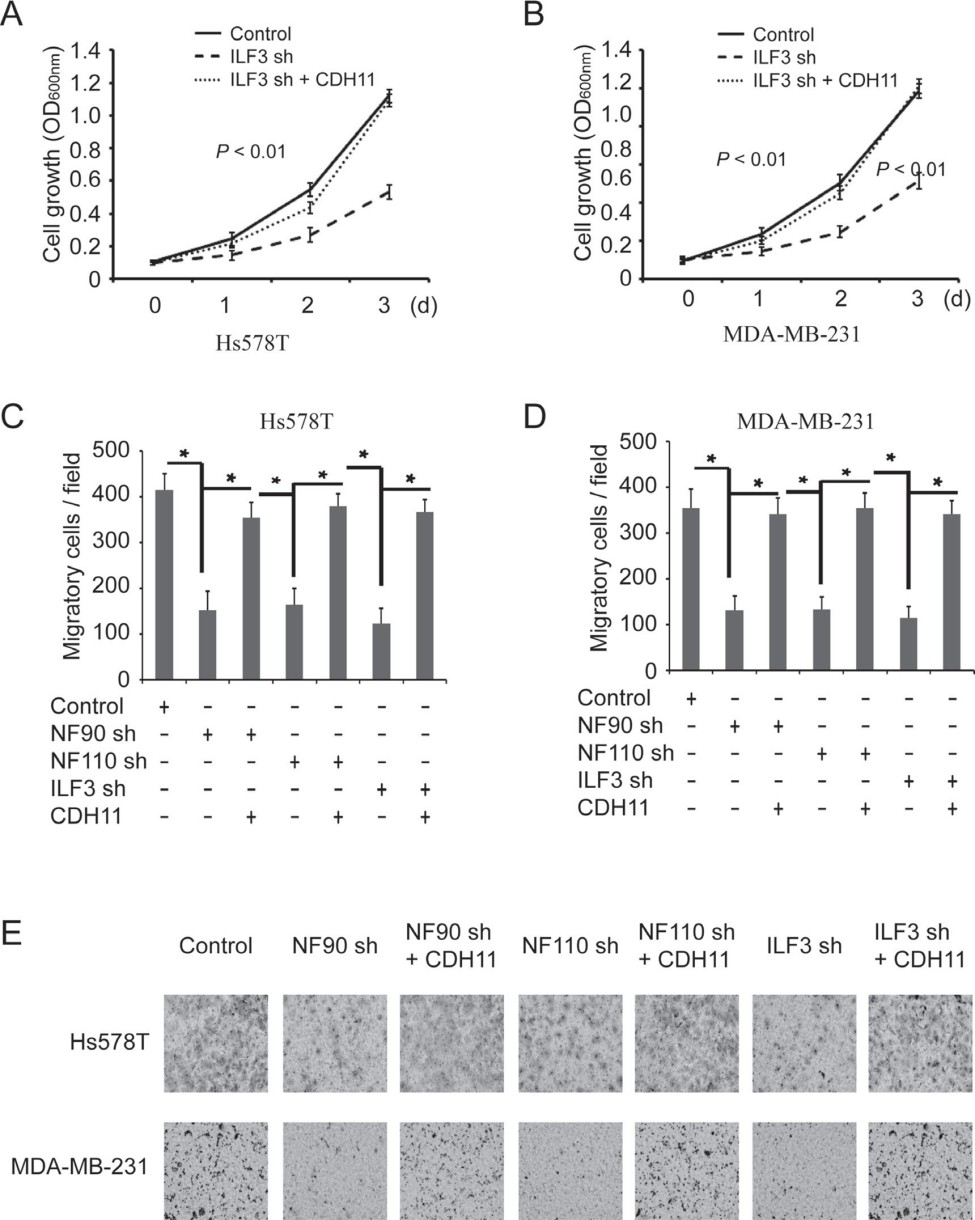
The effects of ILF3 knockdown on cell proliferation and migration are rescued by ecto-expression of CDH11 in breast cancer cell lines (**A** and **B**) ILF3 knockdown cells were lentivirally transduced with CDH11 expression vectors, and MTT assays were performed to analyze cell proliferation. Data are the mean ± SEM; *n* = 3; *P* < 0.01. (**C** and **D**) Hs578T or MDA-MB-231 cells that were transduced with NF90 shRNA, NF110 shRNA or ILF3 shRNA were lentivirally transduced with CDH11 expression vectors, and Transwell assays were performed to analyze cell migration. Columns, mean; bars, SEM; *n* = 3; ^*^P < 0.05. (**E**) Representative images of migratory cells that are crystal violet-stained on the undersurface of transwell.

To determine whether the effects of ILF3 were functionally linked to its regulation of CDH11 transcription, ILF3 knockdown cells were lentivirally transduced with CDH11 expression vectors. MTT assays indicated that ILF3-knockdown breast cancer cells displayed an impaired capability for proliferation, which was almost restored by ectopically expressing CDH11 in the ILF3-knockdown cells (Figure 7A and 7B). Furthermore, ectopic expression of CDH11 largely restored the migration capability of ILF3-knockdown cells (Figure 7C, 7D and 7E). These observations suggested that the effects of ILF3 on breast cancer cells were, at least partially, through its regulation of CDH11 transcription.

### The co-occurrence of CDH11, ILF3 and HOXC8 expression in breast cancer samples

**Figure 8:**
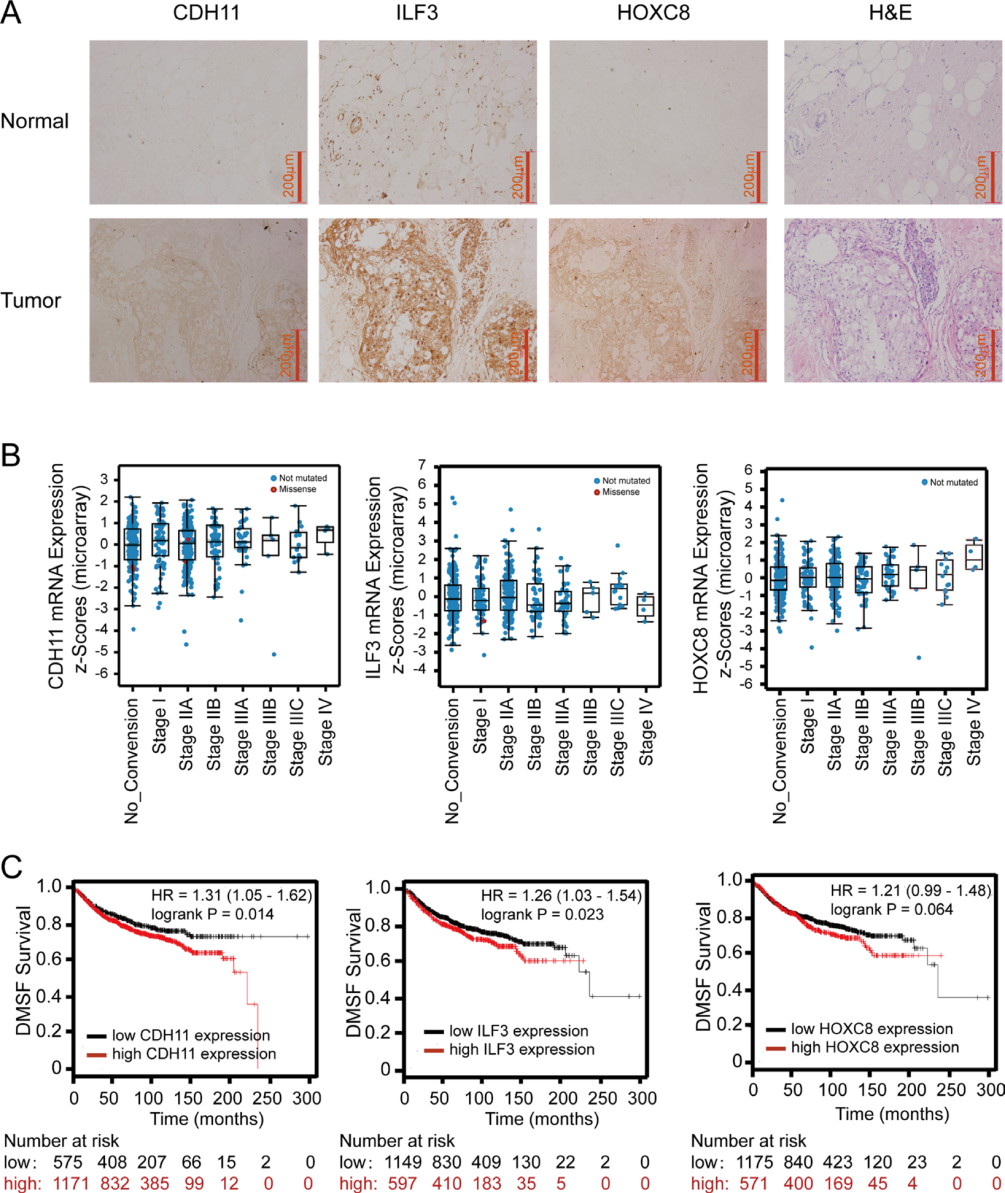
Immunohistochemistry of ILF3, CDH11 and HOXC8 in breast cancer specimens and analyses of ILF3, CDH11 and HOXC8 expression using publicly available datasets (**A**) IHC staining of CDH11, ILF3 and HOXC8 in normal breast and breast cancer tissues. Magnification, 200×; Scale bar, 200 μm. (**B**) mRNA expression levels of CDH11, ILF3 and HOXC8 in breast cancer specimens from different disease stages using the breast cancer dataset (Breast invasive carcinoma, TCGA, Nature 2012, 825 samples) from cBioPortal (www.cbioportal.org). (**C**) Kaplan-Meier survival plots show distant metastasis-free survival for breast cancer patients with high and low expression of CDH11, ILF3 or HOXC8. Data were analyzed using an online survival analysis tool (www.kmplot.com, probe ID: 217804_s_at for ILF3, 207173_x_at for CDH11 and 221350_at for HOXC8).

As a laboratory study may not always recapitulate the clinical malignancy, we performed immunohistochemistry staining (IHC) to examine CDH11, ILF3 and HOXC8 protein levels in breast cancer specimens and normal breast tissues. In breast cancer specimens, more than half of the cases exhibited strong and moderate positivity for CDH11, ILF3 or HOXC8, and only a few cases were negative for CDH11, ILF3 or HOXC8 (7.9% for CDH11, 13.2% for ILF3, and 13.2% for HOXC8). In contrast, none of normal tissues exhibited strong positivity for CDH11, ILF3 or HOXC8, and most cases exhibited no and/or weak positivity. Importantly, the expression differences between the cancer specimens and normal tissues were all statistically significant for CDH11 (*P* < 0.0001), ILF3 (*P* = 0.017) or HOXC8 (*P* = 0.004) (Figure 8A, Supplementary Table 2). These data indicated that breast cancer specimens exhibited higher expression levels of the CDH11, ILF3 and HOXC8 proteins compared to normal breast tissues.

To confirm our IHC results, we explored CDH11, ILF3 and HOXC8 expression levels using publicly available microarray datasets. Online analyses using cBioPortal showed that expression of CDH11, ILF3 or HOXC8 was elevated in the advanced stages of breast cancer, where high mRNA expression levels of CDH11 or HOXC8 were detected in cancer stage IV, and high mRNA expression levels of ILF3 were detected in cancer stage IIIC. However, low mRNA levels of CDH11, ILF3 or HOXC8 were evident in cancer stage 0 (Figure 8B and Supplementary Figure 4). Moreover, expression of CDH11, ILF3 and HOXC8 showed a significant tendency toward co-occurrence among these breast cancer samples (Table 1). Kaplan-Meier survival analyses showed that both high CDH11 and ILF3 were significantly associated with a poor distant metastasis-free survival (DMSF) rate for breast cancer patients (Figure 8C, *P* = 0.014 and 0.023, respectively), and high expression of HOXC8 may also be associated with poor DMSF (*P* = 0.064). More importantly, combination analyses of CDH11, ILF3 and HOXC8 using Kaplan-Meier plotter further showed a poor association for distant metastasis-free survival (*P* = 0.0057) (Supplementary Figure 5). Overall, these results indicated that CDH11, ILF3 and HOXC8 were highly upregulated in the advanced stages of breast cancer, and high expression of CDH11, ILF3 and HOXC8 were associated with a poor DMSF for breast cancer patients.

**Table 1:** Co-occurrence tendency for CDH11, ILF3 and HOXC8 (www.cbioportal.org)

Gene A	Gene B	*p*-value	Log Odds Ratio	Association
CDH11	HOXC8	**< 0.001**	2.234	tendency towards co-occurrence significant
CDH11	ILF3	**< 0.001**	2.216	tendency towards co-occurrence significant
HOXC8	ILF3	**< 0.001**	1.572	tendency towards co-occurrence significant

The query contains no gene pairs with mutually exclusive alteration, and 3 gene pairs with co-occurrent alteration ( 3 significant ) (METABRIC datasets, 2,509 samples, cBioPortal).

## DISCUSSION

The importance of CDH11 in breast cancer progression and metastasis is well established. However, the regulation of CDH11 transcription remains largely unknown. In this study, we demonstrated for the first time that ILF3 interacted with HOXC8 to activate CDH11 transcription in invasive breast cancer cells.

As a member of the HOX family, HOXC8 functions as a transcriptional activator to induce the expression of several genes, including osteopontin (OPN), neural cell adhesion molecule (NCAM), proliferating cell nuclear antigen (PCNA), and CDH11 [33, 34]. It has also been reported that HOXC8 acts as a transcriptional repressor to downregulate the expression of Mgl1, Smad6 and embigin [35–37]. Since CDH11 is expressed only in invasive breast cancer cells and HOXC8 acts as either a transcriptional activator or repressor, we speculated that HOXC8 associated with other proteins to activate CDH11 transcription in breast cancer cells. To test our hypothesis, we performed Co-IP experiment for HOXC8 using CDH11-expressing cells, and observed that ILF3 proteins co-precipitated with HOXC8 proteins by mass spectrometry. Reciprocal Co-IP experiments and immunofluorescence staining confirmed the interaction between HOXC8 and ILF3, and the interaction mainly localized to the nuclei of breast cancer cells (Figure 1). Furthermore, depletion of ILF3 by siRNA knockdown led to significant downregulation of CDH11 expression (Figure 2A and 2B), suggesting that ILF3 was involved in regulating CDH11 expression in breast cancer cells.

The ILF3 family contains four members, namely, NF90a, NF90b, NF110a and NF110b, which are the splice variants of ILF3 transcription [20–22]. In the Co-IP experiments, we found that both NF90a/b and NF110a/b were significantly enriched in the HOXC8 immunoprecipitates, indicating that both NF90 and NF110 interacted with HOXC8 in breast cancer cells. In support of this, individual knockdowns of NF90 or NF110 by shRNA resulted in a decrease in CDH11 expression, as did the ILF3 knockdown, indicating that both NF90 and NF110 were involved in regulating CDH11 expression. This observation was further supported by that the ecto-expression of NF90a, NF90b, NF110a or NF110b greatly enhanced the expression of CDH11 (Figure 2). Therefore, these data showed that all ILF3 members, including NF90a, NF90b, NF110a and NF110b, play important roles in regulation of CDH11 expression.

Studies have shown that ILF3 can regulate gene expression through various mechanisms, such as transcription, translation and mRNA stability [23–25]. We found that ILF3 bound to CDH11 promoter to activate CDH11 transcription, and ILF3 did not affect CDH11 mRNA stability in actinomycin chase experiments, suggesting that ILF3 facilitates CDH11 expression at the transcription level. Moreover, our study showed that HOXC8 and ILF3 co-occupied CDH11 promoter and co-regulated CDH11 transcription. Taken together, our study demonstrated that ILF3 was involved in HOXC8-regulated CDH11 transcription in breast cancer cells (Figures 3, 4 and 5).

It has been reported that overexpression of ILF3 is detected in diverse cancer types, such as nasopharyngeal carcinoma, non-small cell lung cancer and ovarian cancer [29, 31, 38]. In breast cancer, studies have also shown that ILF3 promotes cancer progression by inducing tumor angiogenesis or uPA expression [25, 30]. In this study, we showed that knockdown of ILF3 significantly inhibited proliferation and migration of breast cancer cells, which could be rescued by ecto-expression of CDH11 in ILF3 knockdown cells. This observation suggested that the effects of ILF3 on cell proliferation and migration were, at least partially, due to its induction of CDH11 transcription in breast cancer cells (Figures 6 and 7). Moreover, immunohistochemical staining of breast cancer specimens and normal breast samples showed that the expression of CDH11, ILF3 or HOXC8 was upregulated in breast cancer specimens (Figure 8A). This observation was supported by the meta-analysis (cBioPortal) using publicly available human cancer microarray datasets, in which higher expression levels of CDH11 or HOXC8 were detected in cancer stage IV and that high expression levels of ILF3 were detected in cancer stage IIIC (Figure 8B). The analyses from cBioPortal also showed a significant tendency for co-occurrence between CDH11-HOXC8, CDH11-ILF3 or ILF3-HOXC8 (*P* < 0.001, respectively) (Table 1). More importantly, high expression of CDH11, ILF3 and HOXC8 is linked to poor distant metastasis-free survival (DMFS) for breast cancer patients. In summary, all our data together demonstrated that LF3 and HOXC8 co-regulated CDH11 transcription in breast cancer cells and promoted the proliferation and migration of breast cancer cells by facilitating CDH11 expression in breast cancer cells.

## MATERIALS AND METHODS

### Cells and materials

Human breast cancer cell lines Hs578T and MDA-MB-231 were obtained from American Type Culture Collection and cultured in DMEM supplemented with 10% fetal bovine serum (FBS). Anti-flag M2 (titer, 1:1000) was obtained from Sigma. Anti-ILF3 rabbit (titer, 1:1,000) and anti-β-actin mAb (titer, 1:1,000) were obtained from Santa Cruz Biotechnology. The ILF3 siRNA duplexes were obtained from Thermo Fisher Scientific. TRIzol, Lipofectamine LTX and Lipofectamine 2000 were purchased from Life Technology. All enzymes were purchased from NEB, and all chemicals were purchased from Sigma.

### Co-immunoprecipitation (Co-IP) and mass spectrometry (MS)

Overnight cultured cells were lysed in Co-IP buffer (20 mM Tris at pH 7.4, 0.5% NP40, 150 mM KCl, 1.5 mM MgCl_2_, 0.5 mM dithiothreitol, and protease inhibitors). The cell lysates were precleared with protein G agarose for 1 h at 4°C. The precleared supernatants were then incubated with the specific antibody for 2 h at 4°C, and immune complexes were collected by addition of protein G agarose for 1 h at 4°C. After washing of the beads with Co-IP buffer five times, the immunoprecipitated proteins were eluted with 3 × Flag peptides and separated by SDS-PAGE. The differential band was analyzed by Mass Spectrometry (MS).

### Quantitative RT-PCR

For RNA expression assays, total RNA was extracted using the Trizol reagent (Life Technology). qRT–PCR was used to analyze the expression of CDH11, ILF3 and HOXC8. Target gene expression levels were normalized based on β-actin expression levels. The primers that were used for the qRT–PCR analysis are described in Supplementary Table 1.

### Western blotting analysis

Samples containing equal amounts of protein were separated by SDS–PAGE and electroblotted onto Immobilon-P membranes (Millipore). Western blotting was performed using antibodies as indicated.

### Immunofluorescence staining

Cells grown on glass coverslips were fixed with 4% formaldehyde, permeabilized with 0.2% Triton X-100 and blocked with 3% BSA for 30 min. Cells were incubated with anti-flag M2 antibodies (Sigma) or anti-ILF3 antibodies overnight. After washing, cells were incubated with an Alexa Fluor 488-conjugated anti-mouse antibody (Invitrogen, A11032) and Alexa Fluor 594-conjugated anti-rabbit antibody (Invitrogen, A21207) for 30 min. The nuclei were stained with DAPI. Images were acquired under a confocal laser microscope.

### CDH11 promoter reporters and luciferase assays

The CDH11 promoter sequence was amplified using genomic DNA isolated from MDA-MB-231 cells. The generated CDH11 promoter fragment was subcloned into the pGL4.31 vector (Promega) that contains the firefly luciferase reporter gene. Mutagenesis of the ILF3 binding site was performed by end-prolongation PCR (primer sequences are provided in the Supplemental information). An expression vector encoding Renilla luciferase was included in the transfection for standardization, and a dual luciferase system (Promega) was used to measure luciferase activities according to the manufacturer's protocol.

### Chromatin immunoprecipitation (ChIP) and sequential ChIP (Re-ChIP)

ChIP assays were carried out as previously described [11]. Briefly, the MDA-MB-231 or Hs578T cells were grown to near confluence in 15-cm dishes and fixed in 1% formaldehyde. Sheared chromatin was immunoprecipitated with the anti-HOXC8 antibody overnight at 4°C. Immune complexes were captured using protein G-agarose, and the formaldehyde cross-links in the eluted complexes were reversed. The DNA was analyzed by PCR or real-time PCR. For sequential ChIP, the first ChIP was performed using M2 antibodies in cells transduced with empty vectors or HOXC8-flag expression vectors, followed by a second ChIP with the ILF3 antibodies or negative control immunoglobulin G alone. Pelleted chromatin was treated with RNase, Proteinase K and reverse crosslinked overnight. ChIP'd DNA was extracted with phenol/chloroform and subjected to PCR or Real-time PCR.

### Construction of short hairpin RNA and expression lentiviral vectors

The shRNA sequences for the NF90, NF110 or ILF3 shRNAs were designed as previously described [32] and were subcloned into the pLV-shRNA vector (BioSettia). The NF90a, NF90b, NF110a, NF11b or CDH11 lentiviral expression plasmids were subcloned into pCDH-CMV-MCS-EF1-puro (Biosciences). Lentiviruses were prepared as previously described [12].

### MTT proliferation assay

In total, 30,000 cells per well were seeded into 24-well plates and cultured in media containing 10% fetal bovine serum for 1-3 days. Cell viability was tested by adding 3-(4,5-dimethylthiazol-2-yl)-2,5-diphenyltetrazolium bromide (MTT) solution. After incubating for 4 h at 37°C, the cultured medium was discarded from each well, and 100 μl of dimethyl sulfoxide was added to solubilize the MTT formazan. The color intensity in each plate was read and measured spectrophotometrically using a microplate reader at 560 nm.

### Transwell migration assays

Cell migration was performed as previously described [12]. Briefly, the undersurface of each Transwell chamber (8 μm pore size; Costar) was coated with 10 μg/mL of Collagen I overnight at 4°C. Cells were suspended in serum-free medium at a density of 5 × 10^6^ cells/mL, and 100 μl of the cell suspension was added into each Transwell upper chamber. Meanwhile, 10% FBS was added into the lower chamber to serve as a chemo-attractant. After a 4 h migration period, the remaining cells in the upper chamber were removed with cotton swabs, while the cells on the undersurface of the chamber were stained with a crystal violet solution. The number of migratory cells was determined by counting the stained cells in three different fields under a phase-contrast microscope.

### Clinical samples and immunohistochemistry staining (IHC)

Clinical human breast cancer samples were obtained from Department of Oncology, Tongji Hospital of Huazhong University of Science and Technology. All samples were collected for research use. Paraffin-embedded tissues were sectioned and used for immunohistochemistry with CDH11, HOXC8 and ILF3 as previously described [12]. Sections were also subjected to H&E staining. Staining was scored as +++ if > 75% of the cancer cells were immunostained positive, ++ for 50–75%, + for 25–50%, +/– for 10–25% cells and − if <10% were positive. The staining intensity of each sample was given a modified histochemical score (MH-score) that included an assessment of both the intensity of the stain and the percentage of stained cells [39].

### Bioinformatics analysis

The expression analyses of the CDH11, ILF3 and HOXC8 mRNA levels in the TCGA (Nature 2012, 825 samples) and METABRIC (Nature 2012 & Nat commun 2016, 2,509 samples) patient datasets were performed using cBioPortal (http://www.cbioportal.org) [40]. The Kaplan-Meier analysis was performed using the online Kaplan-Meier Plotter [41] (www.kmplot.com) to estimate distant metastasis-free survival curves, and the best performing threshold was used as the cut-off point for the high and low groups of CDH11, ILF3 and HOXC8 expression. For all statistical analyses, *p* < 0.05 was considered significant.

### Statistical analysis

The data are presented as the means ± s.e.m. Statistical analyses were performed on data collected from at least three independent experiments. The student's *t*-test (two-tailed) was used to compare two groups, and differences were considered statistically significant when *p* < 0.05. Statistical analyses were performed with GraphPad Prism with significance levels set at ^*^P < 0.05 and ^**^P < 0.01.

## SUPPLEMENTARY MATERIALS FIGURES AND TABLES



## Abbreviations

ILF3: Interleukin Enhancer-Binding Factor 3
HOXC8: Homeobox C8
CDH11: cadherin 11
Co-IP: co-immunoprecipitation
MS: mass spectrometry
IHC: immunohistochemistry
ChIP: Chromatin immunoprecipitation
EMT: epithelial-to-mesenchymal transition
DMSF: distant metastasis-free survival

## References

[1] Thiery JP (2002). Epithelial-mesenchymal transitions in tumour progression. Nat Rev Cancer.

[2] Hazan RB, Qiao R, Keren R, Badano I, Suyama K (2004). Cadherin switch in tumor progression. Ann N Y Acad Sci.

[3] Okazaki M, Takeshita S, Kawai S, Kikuno R, Tsujimura A, Kudo A, Amann E (1994). Molecular cloning and characterization of OB-cadherin, a new member of cadherin family expressed in osteoblasts. J Biol Chem.

[4] Simonneau L, Kitagawa M, Suzuki S, Thiery JP (1995). Cadherin 11 expression marks the mesenchymal phenotype: towards new functions for cadherins?. Cell Adhes Commun.

[5] Pishvaian MJ, Feltes CM, Thompson P, Bussemakers MJ, Schalken JA, Byers SW (1999). Cadherin-11 is expressed in invasive breast cancer cell lines. Cancer Res.

[6] Huang CF, Lira C, Chu K, Bilen MA, Lee YC, Ye X, Kim SM, Ortiz A, Wu FL, Logothetis CJ, Yu-Lee LY, Lin SH (2010). Cadherin-11 increases migration and invasion of prostate cancer cells and enhances their interaction with osteoblasts. Cancer Res.

[7] Deng Z, Niu G, Cai L, Wei R, Zhao X (2013). The prognostic significance of CD44V6, CDH11, and beta-catenin expression in patients with osteosarcoma. Biomed Res Int.

[8] Braungart E, Schumacher C, Hartmann E, Nekarda H, Becker KF, Hofler H, Atkinson MJ (1999). Functional loss of E-cadherin and cadherin-11 alleles on chromosome 16q22 in colonic cancer. J Pathol.

[9] Shibata T, Ochiai A, Gotoh M, Machinami R, Hirohashi S (1996). Simultaneous expression of cadherin-11 in signet-ring cell carcinoma and stromal cells of diffuse-type gastric cancer. Cancer Lett.

[10] Shimazui T, Giroldi LA, Bringuier PP, Oosterwijk E, Schalken JA (1996). Complex cadherin expression in renal cell carcinoma. Cancer Res.

[11] Li Y, Chao F, Huang B, Liu D, Kim J, Huang S (2014). HOXC8 promotes breast tumorigenesis by transcriptionally facilitating cadherin-11 expression. Oncotarget.

[12] Li Y, Zhang M, Chen H, Dong Z, Ganapathy V, Thangaraju M, Huang S (2010). Ratio of miR-196s to HOXC8 messenger RNA correlates with breast cancer cell migration and metastasis. Cancer Res.

[13] Li Y, Guo Z, Chen H, Dong Z, Pan ZK, Ding H, Su SB, Huang S (2011). HOXC8-Dependent Cadherin 11. Expression Facilitates Breast Cancer Cell Migration through Trio, Rac. Genes Cancer.

[14] Lei H, Wang H, Juan AH, Ruddle FH (2005). The identification of Hoxc8 target genes. Proc Natl Acad Sci U S A.

[15] Adwan H, Zhivkova-Galunska M, Georges R, Eyol E, Kleeff J, Giese NA, Friess H, Bergmann F, Berger MR (2011). Expression of HOXC8 is inversely related to the progression and metastasis of pancreatic ductal adenocarcinoma. Br J Cancer.

[16] Alami Y, Castronovo V, Belotti D, Flagiello D, Clausse N (1999). HOXC5 and HOXC8 expression are selectively turned on in human cervical cancer cells compared to normal keratinocytes. Biochem Biophys Res Commun.

[17] Axlund SD, Lambert JR, Nordeen SK (2010). HOXC8 inhibits androgen receptor signaling in human prostate cancer cells by inhibiting SRC-3 recruitment to direct androgen target genes. Mol Cell Biol.

[18] Gehring WJ, Hiromi Y (1986). Homeotic genes and the homeobox. Annu Rev Genet.

[19] Gehring WJ (1985). Homeotic genes, the homeobox, and the spatial organization of the embryo. Harvey Lect.

[20] Duchange N, Pidoux J, Camus E, Sauvaget D (2000). Alternative splicing in the human interleukin enhancer binding factor 3 (ILF3) gene. Gene.

[21] Reichman TW, Parrott AM, Fierro-Monti I, Caron DJ, Kao PN, Lee CG, Li H, Mathews MB (2003). Selective regulation of gene expression by nuclear factor 110, a member of the NF90 family of double-stranded RNA-binding proteins. J Mol Biol.

[22] Chaumet A, Castella S, Gasmi L, Fradin A, Clodic G, Bolbach G, Poulhe R, Denoulet P, Larcher JC (2013). Proteomic analysis of interleukin enhancer binding factor 3 (Ilf3) and nuclear factor 90 (NF90) interactome. Biochimie.

[23] Kiesler P, Haynes PA, Shi L, Kao PN, Wysocki VH, Vercelli D (2010). NF45 and NF90 regulate HS4-dependent interleukin-13 transcription in T cells. J Biol Chem.

[24] Kuwano Y, Kim HH, Abdelmohsen K, Pullmann R, Martindale JL, Yang X, Gorospe M (2008). MKP-1 mRNA stabilization and translational control by RNA-binding proteins HuR and NF90. Mol Cell Biol.

[25] Vumbaca F, Phoenix KN, Rodriguez-Pinto D, Han DK, Claffey KP (2008). Double-stranded RNA-binding protein regulates vascular endothelial growth factor mRNA stability, translation, and breast cancer angiogenesis. Mol Cell Biol.

[26] Sakamoto S, Aoki K, Higuchi T, Todaka H, Morisawa K, Tamaki N, Hatano E, Fukushima A, Taniguchi T, Agata Y (2009). The NF90-NF45 complex functions as a negative regulator in the microRNA processing pathway. Mol Cell Biol.

[27] Shamanna RA, Hoque M, Lewis-Antes A, Azzam EI, Lagunoff D, Pe’ery T, Mathews MB (2011). The NF90/NF45 complex participates in DNA break repair via nonhomologous end joining. Mol Cell Biol.

[28] Jiang W, Huang H, Ding L, Zhu P, Saiyin H, Ji G, Zuo J, Han D, Pan Y, Ding D, Ma X, Zhang Y, Wu J (2015). Regulation of cell cycle of hepatocellular carcinoma by NF90 through modulation of cyclin E1 mRNA stability. Oncogene.

[29] Guo Y, Fu P, Zhu H, Reed E, Remick SC, Petros W, Mueller MD, Yu JJ (2012). Correlations among ERCC1, XPB, UBE2I, EGF, TAL2 and ILF3 revealed by gene signatures of histological subtypes of patients with epithelial ovarian cancer. Oncol Rep.

[30] Hu Q, Lu YY, Noh H, Hong S, Dong Z, Ding HF, Su SB, Huang S (2013). Interleukin enhancer-binding factor 3 promotes breast tumor progression by regulating sustained urokinase-type plasminogen activator expression. Oncogene.

[31] Guo NL, Wan YW, Tosun K, Lin H, Msiska Z, Flynn DC, Remick SC, Vallyathan V, Dowlati A, Shi X, Castranova V, Beer DG, Qian Y (2008). Confirmation of gene expression-based prediction of survival in non-small cell lung cancer. Clin Cancer Res.

[32] Guan D, Altan-Bonnet N, Parrott AM, Arrigo CJ, Li Q, Khaleduzzaman M, Li H, Lee CG, Pe’ery T, Mathews MB (2008). Nuclear factor 45 (NF45) is a regulatory subunit of complexes with NF90/110 involved in mitotic control. Mol Cell Biol.

[33] Lei H, Juan AH, Kim MS, Ruddle FH (2006). Identification of a Hoxc8-regulated transcriptional network in mouse embryo fibroblast cells. Proc Natl Acad Sci U S A.

[34] Min H, Lee JY, Bok J, Chung HJ, Kim MH (2010). Proliferating cell nuclear antigen (Pcna) as a direct downstream target gene of Hoxc8. Biochem Biophys Res Commun.

[35] Chao F, Zhang J, Zhang Y, Liu H, Yang C, Wang J, Guo Y, Wen X, Zhang K, Huang B, Liu D, Li Y (2015). Embigin, regulated by HOXC8, plays a suppressive role in breast tumorigenesis. Oncotarget.

[36] Ruthala K, Gadi J, Lee JY, Yoon H, Chung HJ, Kim MH (2011). Hoxc8 downregulates Mgl1 tumor suppressor gene expression and reduces its concomitant function on cell adhesion. Mol Cells.

[37] Kang M, Bok J, Deocaris CC, Park HW, Kim MH (2010). Hoxc8 represses BMP-induced expression of Smad6. Mol Cells.

[38] Fung LF, Lo AK, Yuen PW, Liu Y, Wang XH, Tsao SW (2000). Differential gene expression in nasopharyngeal carcinoma cells. Life Sci.

[39] McCarty KS, McCarty KS (1984). Histochemical approaches to steroid receptor analyses. Semin Diagn Pathol.

[40] Gao J, Aksoy BA, Dogrusoz U, Dresdner G, Gross B, Sumer SO, Sun Y, Jacobsen A, Sinha R, Larsson E, Cerami E, Sander C, Schultz N (2013). Integrative analysis of complex cancer genomics and clinical profiles using the cBioPortal. Sci Signal.

[41] Gyorffy B, Lanczky A, Eklund AC, Denkert C, Budczies J, Li Q, Szallasi Z (2010). An online survival analysis tool to rapidly assess the effect of 22,277 genes on breast cancer prognosis using microarray data of 1,809 patients. Breast Cancer Res Treat.

