# Supplementary material for: Interleukin enhancer-binding factor 3 and HOXC8 co-activate cadherin 11 transcription to promote breast cancer cells proliferation and migration
Source: Oncotarget. 2017 Nov 18;8(64):107477–91. doi: 10.18632/oncotarget.22491 (PMC5746082; doi:10.18632/oncotarget.22491)
Supplement: Supplementary file 1 [file oncotarget-08-107477-s001.pdf]

Interleukin enhancer-binding factor 3 and HOXC8 co-activate cadherin 11 transcription to promote breast cancer cells proliferation and migration

SUPPLEMENTARY MATERIALS

Supplementary Table 1: Oligonucleotides used in real-time PCR, cloning and knockdown studies

Primer Name	Sequence (5′ to 3′)
CDH11 promoter cloning	
Forward	5′-AAGCTAGCTCCAGTTCATATAAATGGCCATTTCATATA-3′
Reverse	5′-AAGATATCGTCCCGGTCCCATTCACAAGTCAGCGGCGG-3′
ChIP primers	
Forward-1	5′-TTAGAGCATTTGATTGC-3′
Reverse-1	5′-TATATGAACTGGAAAGGC-3′
Forward-2	5′-TTCTTCCCCTTTTATTGC-3′
Reverse-2	5′-CCATCAACTGGTGAGCAG-3′
Forward-3	5′-TGGGTATTTGTGTTGTTTC-3′
Reverse-3	5′-CCAGCAAGTTAGGATTCT-3′
Forward-4	5′-TGATTCAGGTGTGTAGTG-3′
Reverse-4	5′-TTATCGAATCACAACAGT-3′
Knockdown shRNA	
Scrambled shRNA	5′-CAACAAGATGAAGAGCACCAATT-3′
HOXC8 shRNA	5′-GCAATATCCCGACTGTAAATCTT-3′
ILF3 shRNA	5′-GTGCTGGTTCCAACAAAA-3′
NF 90 shRNA	5′-CAGACTGCTACGGCTATCA-3′
NF110 shRNA	5′-CTACGAGAGCAAATTCAAC-3′
mutagenesis	
Forward 1	GACCTTGGGCTGGTTGGTTGGATATACTGT
Reverse 1	TGGATTAACTGCAGGCCAAATCCCCAC
Forward 2	CAGTTAATCCAACCTCTCTCTGCTTGCAGAAG
Reverse 2	AAGGGCCCTTTTGGTTACGTGGTAGGCACAGGAGAATG
Real-time PCR	
Cadherin 11 forward	5′-GCATCCCGCCCATGAGTA-3′
Cadherin 11 reverse	5′-CGCACCCGCAGACTTTG-3′
ILF3 forward	5′-ACAGCAACGGGAAGATATCAC-3′
ILF3 reverse	5′-CCACTGGGTTTTCATTCTTTGG-3′
β-actin forward	5′-TGGATCAGCAAGCAGGAGTATG-3′
β-actin reverse	5′-GCATTTGCGGTGGACGAT-3′

Supplementary Table 2: IHC staining of CDH11, ILF3 and HOXC8 expression

	Tumor tissues (*n* = 38)	Normal tissues (*n* = 10)	*P*-value
CDH11, *n* (%)			
0	3 (7.9)	7 (70)	
+	10 (26.3)	3 (30)	< 0.0001
++	15 (39.5)	0	
+++	10 (26.3)	0	
ILF3			
0	5 (13.2)	4 (40)	
+	12 (31.6)	4 (40)	0.017
++	14 (36.8)	2 (20)	
+++	7 (18.4)	0	
HOXC8			
0	5 (13.2)	5 (50)	
+	10 (26.3)	3 (30)	0.004
++	12 (31.6)	2 (20)	
+++	11 (29)	0	

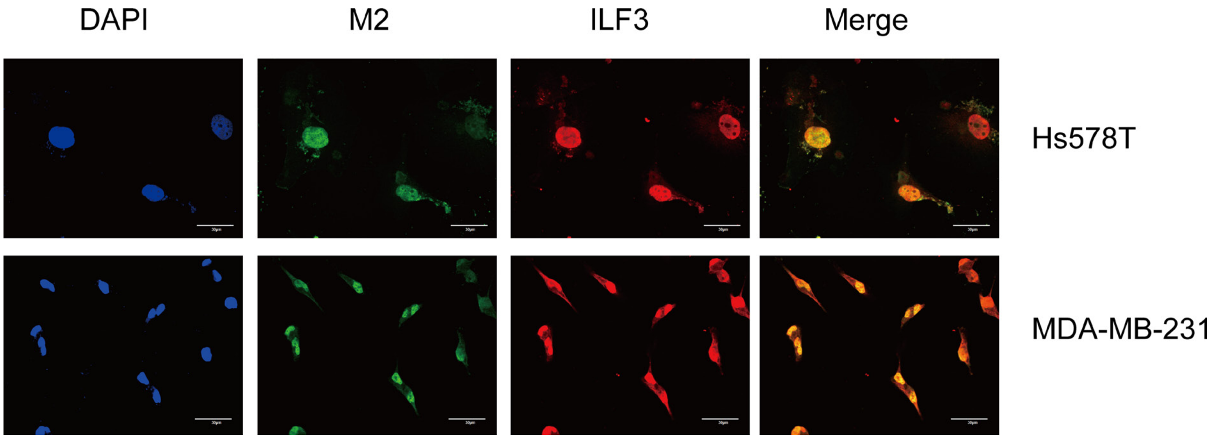

Supplementary Figure 1: Co-localization of HOXC8 and ILF3 in Hs578T and MDA-MB-231 cells. Immunofluorescence staining for HOXC8-flag (green) and ILF3 (red) in Hs578T or MDA-MB-231 cells lentivirally transduced with expression vectors encoding HOXC8-flag; DAPI staining for the nucleus. Magnification, 600×; Scale bar, 30 µm.

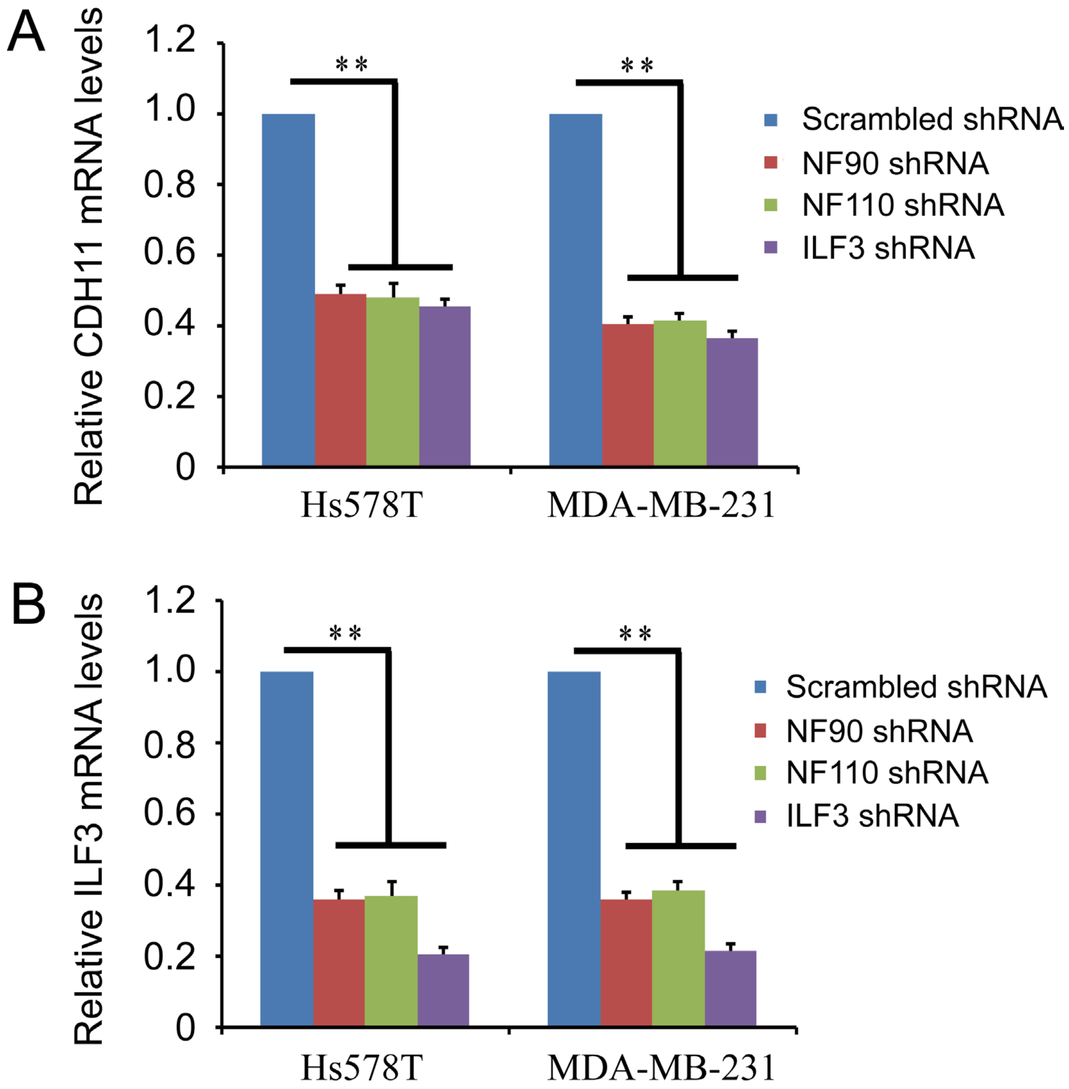

Supplementary Figure 2: Depletion of NF90, NF110 or ILF3 results in reduction of CDH11 mRNA levels. Hs578T or MDA-MB-231 cells were lentivirally transduced with NF90, NF110 or ILF3-specific shRNA, and total RNA was isolated and subjected to qRT-PCR to examine the level of CDH11 mRNA (**A**) or ILF3 mRNA (**B**). β-actin mRNA was used as an internal control for standardization. Each sample was run in triplicate and in multiple experiments for mean ± SEM; $^{**}P < 0.01$.

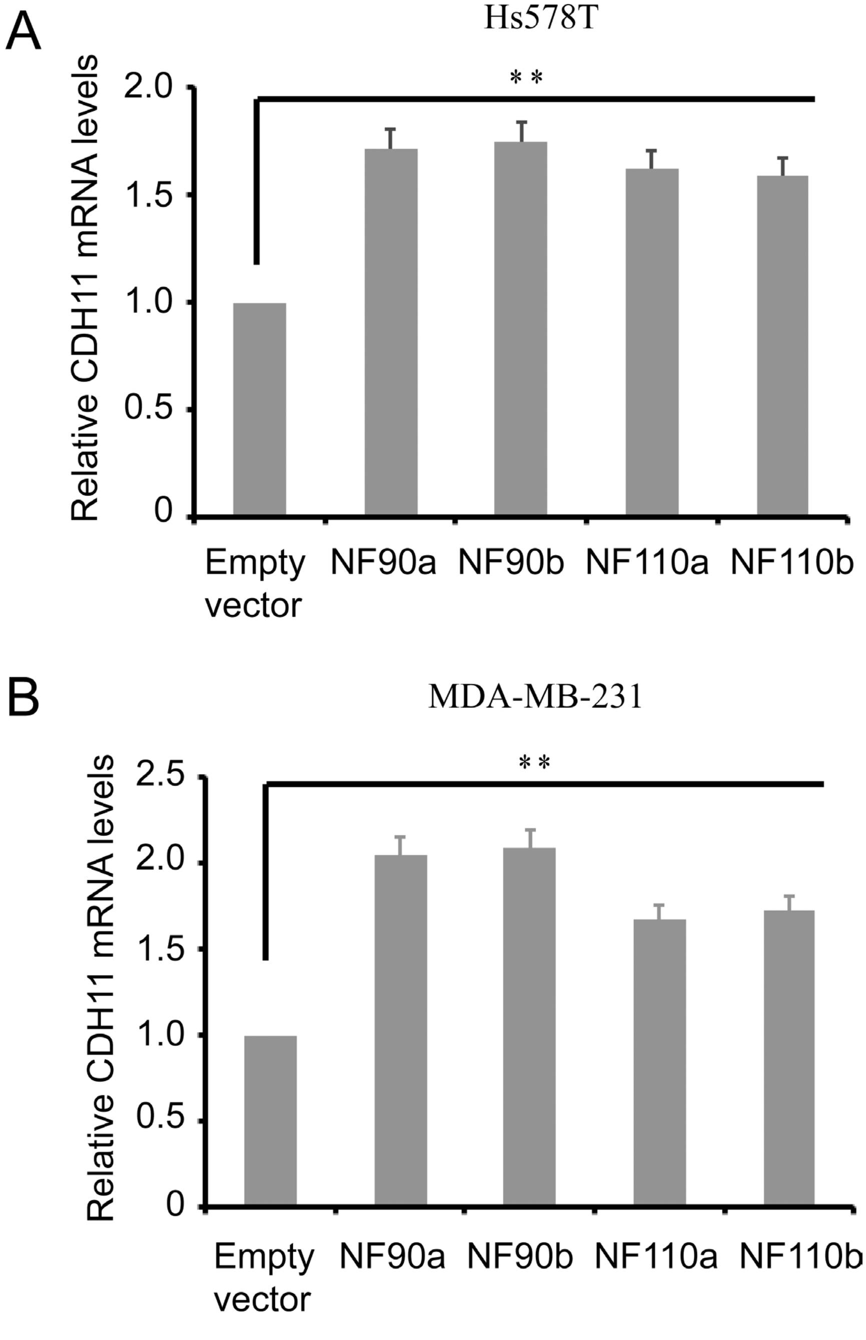

Supplementary Figure 3: Ecto-expression of NF90a, NF90b, NF110a or NF110b increases CDH11 mRNA levels. Hs578T or MDA-MB-231 cells were lentivirally transduced with NF90a, NF90b, NF110a or NF110b expression vectors, and qRT-PCR was performed to examine the levels of CDH11 mRNA. β-actin mRNA was used as an internal control for standardization. Each sample was run in triplicate and in multiple experiments for mean ± SEM; $^{**}P < 0.01$.

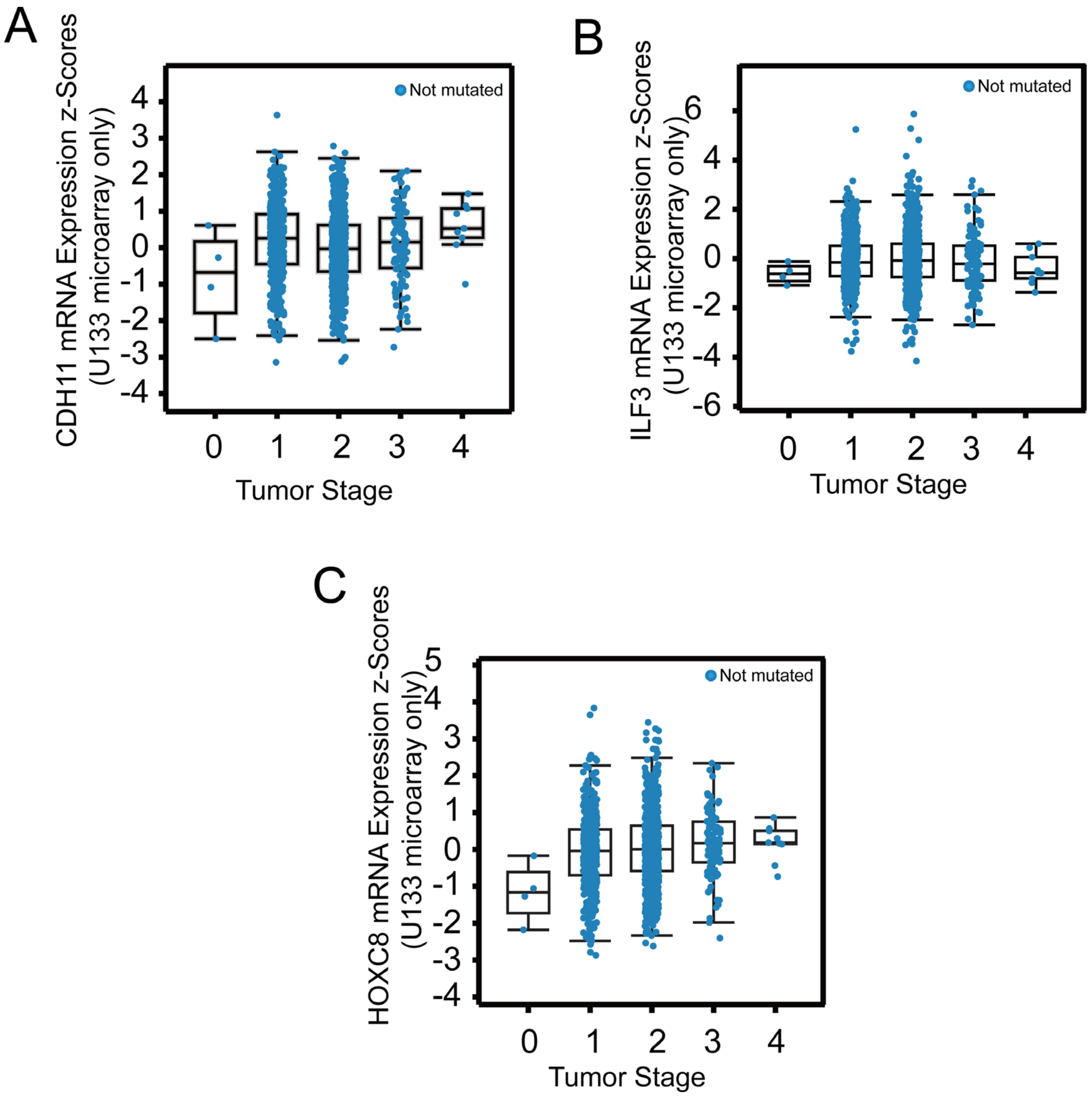

Supplementary Figure 4: Meta-analysis of CDH11, ILF3 and HOXC8 mRNA levels using publicly available human cancer microarray datasets. mRNA expression levels of CDH11 (**A**), ILF3 (**B**) and HOXC8 (**C**) at different stages of breast tumor specimen using breast cancer dataset (METABRIC, Nature 2012 & Nat Commun 2016, 2509 samples) from cBioPortal (www.cbioportal.org).

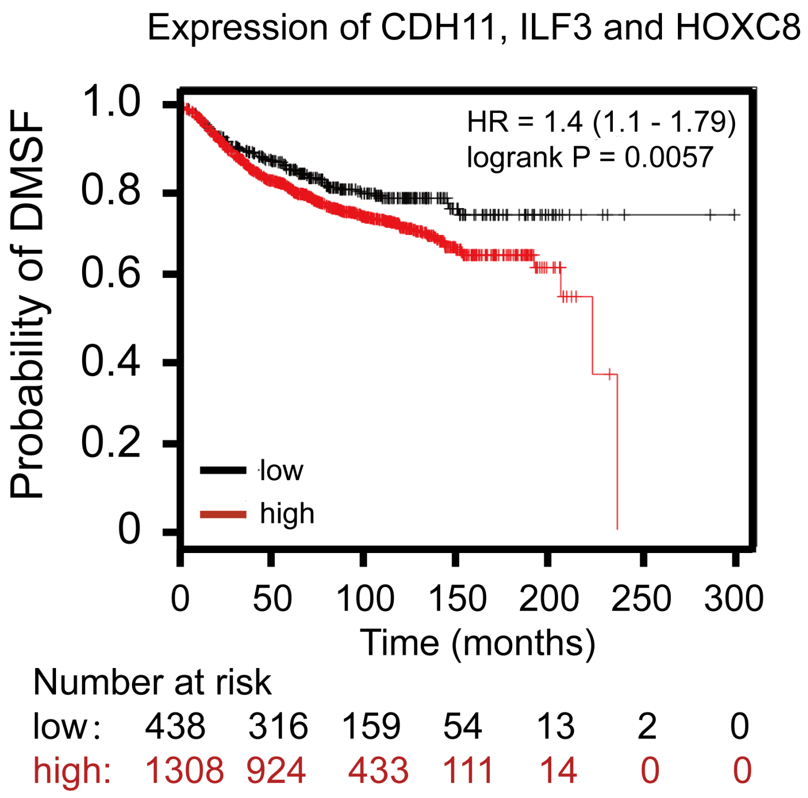

Supplementary Figure 5: Association of CDH11, HOXC8 and ILF3 with DMSF in breast cancer patients. The combined analyses of CDH11 + HOXC8 + ILF3 showed poor association for distant metastasis-free survival probability (DMSF). Data were analyzed using online survival analysis tool (www.kmplot.com) (probe ID: 217804_s_at for ILF3, 207173_x_at for CDH11 and 221350_at for HOXC8).
